# Epizootic Cellulitis among Horses

**Published:** 1899-08

**Authors:** 


EPIZOÖTIC CELLULITIS AMONG HORSES. Dr. Manley, of Pittsburg, Kansas, reports an epizoötic among horses for the past ten months in that section of the State, with very serious results. Early in the attack they sweat profusely, later the skin becomes dry and parched, remaining so until death. Gradual loss of appetite for all good, wholesome food, but a depraved appetite later on, seeking coarse, woody hay or vegetable fibre, their bedding, and pulling up grass by the roots. It attacks all ages of the equine species from three months old up; mules and asses are exempt. A slight cough is at times present in the early stages, more especially among the old stock. They assume the recumbent position for but short periods at a time, and are uneasy at times, evinced by stamping a hind foot on the ground. In several mares I have noticed loss of control of the hind-parts, and a winding or weaving. At times they will rest the head against the side of the stall or in a corner, keeping it down low. One case under observation for months ate nothing but the coarsest provender and green corn. Temperature fluctuates between 98° to 102½°. Dr. Berry, of Joplin, Missouri, has designated the diseases as epizoötic cellulitis, and advised the use of acetanilid, quinine, whiskey, and cinchona, but with no noticeable benefit. They usually die from exhaustion, with extreme emaciation.

Dr. Manley would like to hear from other veterinarians in Kansas or elsewhere.

